# Growth Performance and Plasma Metabolites of Grazing Beef Cattle Backgrounded on Buffel or Buffel-*Desmanthus* Mixed Pastures

**DOI:** 10.3390/ani11082355

**Published:** 2021-08-09

**Authors:** Felista W. Mwangi, Christopher P. Gardiner, Glen Walker, Trevor J. Hall, Bunmi S. Malau-Aduli, Robert T. Kinobe, Aduli E. O. Malau-Aduli

**Affiliations:** 1 Animal Genetics and Nutrition, Veterinary Sciences Discipline, College of Public Health, Medical and Veterinary Sciences, Division of Tropical Health and Medicine, James Cook University, Townsville, QLD 4811, Australia; felista.mwangi@my.jcu.edu.au (F.W.M.); christopher.gardiner@jcu.edu.au (C.P.G.); glen.walker@jcu.edu.au (G.W.); robert.kinobe@jcu.edu.au (R.T.K.); 2 Hallmark Rural Consulting, 75 Love Road, Vale View, QLD 4352, Australia; trevhall02@yahoo.com.au; 3 College of Medicine and Dentistry, Division of Tropical Health and Medicine, James Cook University, Townsville, QLD 4811, Australia; bunmi.malauaduli@jcu.edu.au

**Keywords:** plasma metabolites, buffel grass, tropical beef cattle, growth performance, tropical pastures, backgrounding, legumes, liveweight

## Abstract

**Simple Summary:**

Pasture quality and digestibility decline during the dry season resulting in weight loss or marginal weight gains of grazing cattle in the seasonally dry subtropics of northern Australia. Oversowing grass with legume pastures has shown potential to improve pasture quality and cattle weight gain. This study aimed to evaluate the change in steers’ weight gain and plasma metabolites in response to grazing buffel grass pastures oversown with *Desmanthus* spp. (*Desmanthus*), a tropical legume adapted to cracking clay soils, compared to buffel-grass-only pastures. Results showed that *Desmanthus* at a low botanical composition had no effect on weight gain and plasma metabolites, although pasture yield and stocking rate were 443 kg/ha and 9.5% higher, respectively. Since the productivity of grazing systems depends on cattle annual weight gain and stocking rate, the practical implication of this study is that *Desmanthus* may improve the profitability of beef production in the dry tropics of northern Australia by improving pasture-carrying capacity with no adverse effect on cattle health status and growth performance.

**Abstract:**

Dietary crude protein and dry matter digestibility are among the major factors limiting feed intake and weight gain of cattle grazing native and improved pastures in the subtropics of Northern Australia during the dry season. Incorporating a suitable legume into grasses improves pasture quality and cattle weight gain, but only a limited number of legume pastures can establish and persist in cracking clay soils. This study aimed to evaluate the effect of *Desmanthus* inclusion in buffel grass (*Cenchrus ciliaris*) pastures on the plasma metabolite profile and growth performance of grazing beef cattle during the dry season. We hypothesised that backgrounding steers on buffel grass-*Desmanthus* mixed pastures would elicit significant changes in plasma glucose, bilirubin, creatinine, non-esterified fatty acids and β-hydroxybutyrate, resulting in higher liveweight gains than in steers on buffel grass only pastures. Four hundred tropical composite steers were assigned to buffel grass only (*n* = 200) or buffel grass oversown with *Desmanthus* (11.5% initial sward dry matter) pastures (*n* = 200) and grazed for 147 days during the dry season. *Desmanthus* accounted for 6.2% sward dry matter at the end of grazing period. Plasma metabolites results showed that changes in β-hydroxybutyrate, creatinine, bilirubin, glucose and non-esterified fatty acids were within the expected normal range for all the steers, indicating that with or without *Desmanthus* inclusion in the diet of grazing steers, animal health status was not compromised. It was also evident that *Desmanthus* inclusion in buffel grass pastures had no impact on the plasma metabolite profile, liveweight and daily weight gain of grazing steers. Therefore, our tested hypothesis of higher changes in plasma metabolite profile and higher liveweight gains due to backgrounding on low-level buffel grass-*Desmanthus* mixed pastures does not hold.

## 1. Introduction

Livestock production in the tropics plays a significant role in terms of animal numbers, total products output and employment globally [1], but beef cattle production measured as annual live weight gain is low from tropical pastures compared to temperate pastures [2]. In northern Australia’s dry tropical environment, beef cattle rely mainly on extensive grazing of unimproved native pastures dominated by C4 grasses with limited use of exotic pasture species [3,4,5]. The dry tropics are characterized by a distinct wet and dry season, both of which vary greatly in length; for instance, the dry season varies from four to nine months of the year [6]. As a result, the quantity and nutritive value of pastures vary widely throughout the year. Pasture growth takes place in the wet season of November to April, resulting in increased green herbage mass, crude protein content and dry matter digestibility. Towards the end of wet/growing season and during the dry season, pasture senescence reduces green herbage mass, crude protein content, dry matter digestibility and, consequently, cattle dry matter intake [7,8]. Thus, high cattle weight gains are observed during the wet season, which can exceed a kilogram per day [6], but reduces in the dry season, sometimes resulting in weight loss [9].

The importance of tropical legume pastures to improve beef production has long been established [10,11,12]. The integration of legumes into grass pastures increases protein and digestible energy intake resulting in improved cattle growth rate and reduced age at slaughter [9]. In northern Australia, pasture legumes came to general use over five decades ago [13] and legumes of the genus *Stylosanthes* (Stylo) have a significant economic impact on light soils of tropical northern Australia [14,15], but there was no suitable legume pasture for the regions with cracking clay (vertosol) soils until recently [16]. Vertosol soils play a significant role in northern Australian beef cattle production, particularly in the State of Queensland, which accounts for 46% of the Australian beef cattle herd [17]. Vertosol soils also occupy 28% of the total area [18] and account for over 3.2 million ha of land [19] within the subcoastal north-eastern Australia between latitudes 16° S and 25° S [15].

Legumes of the genus *Desmanthus* spp. (referred to as *Desmanthus* henceforth) can be utilised for pasture improvement. *Desmanthus* persists on cracking clay soils, grows in a wide range of rainfall zones, survives in as low as 400 mm of rainfall per annum, is highly productive [20,21] and decreases methane emissions in beef cattle [22,23]. Hill et al. [24] reported an increase in the use of legume-based pastures for livestock production in Australia due to financial pressure that has prompted the need for a more cost-effective protein source. As a result, over 35,000 ha of the three commercially available *Desmanthus* species (*D. bicornutus*, *D. leptophyllus* and *D. virgatus*), have been established across many regions of Australia including Queensland, Northern Territory and northern New South Wales since 2012 [25]. However, only limited literature exists on the effect of *Desmanthus* pasture grazing on animal growth performance and none on plasma metabolites profile. A study on the effect of *Desmanthus* on steer performance reported that steers grazing *Desmanthus*/buffel grass pastures were 30 kg heavier than those grazing buffel-grass-only pastures after 90 days [26]. Goats fed *Brachiaria mulato* (Mulato) grass and supplemented with *Desmanthus* at 27% dry matter intake (DMI) gained 17 g/day more than those fed Mulato grass only [27]. Supplementing sheep fed Mitchell grass (*Astrebla* spp.) basal diet with *D. leptophyllus*, *D. pubescens* or *D. virgatus* hay reduced weight loss from 5.83 kg/hd in control to between 1.33 and 2.33 kg/hd [28]. In contrast, growing goats fed *Sorghum bicolor* (Sudan grass) and supplemented with *D. bicornutus* at 40% DMI gained 16 g/day less weight compared to those supplemented with Leucaena, alfalfa and lablab [29]. These studies were either indoor trials or conducted in small paddocks, which do not represent the extensive grazing systems of northern Australia. In addition, pasture legume levels of 27–40% used in these indoor studies may not be achieved.

Liveweight and body condition scores are traditional routine methods used to evaluate cattle nutritional status because they are quicker to perform and requires less expertise, but they are associated with several limitations [30]. Bodyweight evaluates nutritional status by measuring growth as a function of cell enlargement, cell multiplication and incorporation of constituents from the environment, for example, in apatite deposition [31]. Change in body weight can result from tissue hydration, change in gut and bladder fill, pregnancy and parturition rather than change in body fat or protein content [32]. Body condition score assesses the animal nutritional status over time as a function of the level of fatness on the animal [33], but is less reliable due to the general subjective nature [30]. Plasma metabolites, on the other hand, provide an integrated index of nutrient supply adequacy in relation to nutrient utilisation [34] and provide an immediate indication of the animal’s present nutritional status [35]. Animals grazing low-quality pastures during the dry season mobilize fatty acids from the adipose tissue as a long-term response to the negative energy balance resulting in elevated NEFA and BHB [36,37]. Supplementing animals fed low-quality grass diet with legumes improves their nutritional plane, thus minimizing catabolism to encourage anabolic processes. In addition to improving the nutritional plane of animals, legume supplementation improves their health status. Supplementing grass-fed sheep with *Moringa oleifera* was reported to increase blood glucose and immunoglobulin A levels [10]. In another study, calves supplemented with alfalfa hay had lower plasma BHB compared to their unsupplemented counterparts [38]. Although numerous reports on the effect of dietary legume supplementation on blood parameters in dairy cows exist [39,40], little information is reported on beef cattle [41]. Therefore, the primary aim of this study was to evaluate the growth performance and plasma metabolites of beef cattle backgrounded (the grazing period between weaning and finishing) on buffel grass pasture oversown with *Desmanthus* during the dry season. We hypothesised that backgrounding steers on low-level buffel grass-*Desmanthus* mixed pastures would elicit significant changes in plasma glucose, bilirubin, creatinine, non-esterified fatty acids and β-hydroxybutyrate, resulting in higher liveweight gains than in steers on buffel-grass-only pastures.

## 2. Materials and Methods

All procedures in this study followed the James Cook University Animal Ethics Committee approved guidelines (Approval Number 2639) in accordance with the Australian code of practice for the care and use of animals for scientific purposes [42].

### 2.1. Study Site

This dry season on-farm study from 9th July to 3rd December 2019 was carried out at Cungelella, a commercial beef pastoral property in central Queensland (24°41′ S, 147°10′ E), Australia. The mean annual rainfall of the farm is 598 mm with mean minimum and maximum temperatures of 12.7 °C and 29.1 °C, respectively. The soils are typically low in nitrogen and phosphorus, alkaline and contain moderate to high clay content [43]. Two buffel grass-dominated paddocks were assigned as buffel grass (575 ha) and mixed buffel grass-*Desmanthus* (520 ha) pastures. *Desmanthus* was sown in March 2018 in established buffel grass pastures. The paddock was sprayed with glyphosate-based herbicide (Roundup; Monsanto, Kilda Road, Melbourne, Australia) at the rate of 3 in 37 L (*v*/*v*) of water per ha and then *Desmanthus* seed was aerial-sown at the rate of 3–5 kg/ha. *Desmanthus* (Progardes^®^; Agrimix Pastures Pty Ltd., Ferny Hills DC, QLD, Australia) was a blend of *D. leptophyllus*, *D. virgatus* and *D. bicornutus* (cultivars JCU2, JCU4, JCU5 and JCU7), which range from early, medium to late maturing species [44,45]. The pastures were not fertilised. After self-seeding re-establishment of buffel grass, both paddocks were grazed heavily in 2018 to control competition and for *Desmanthus* to establish well [46]. The paddocks were destocked in September 2019, before the start of the wet season that usually starts in November.

### 2.2. Animal Management

**Figure 1:**
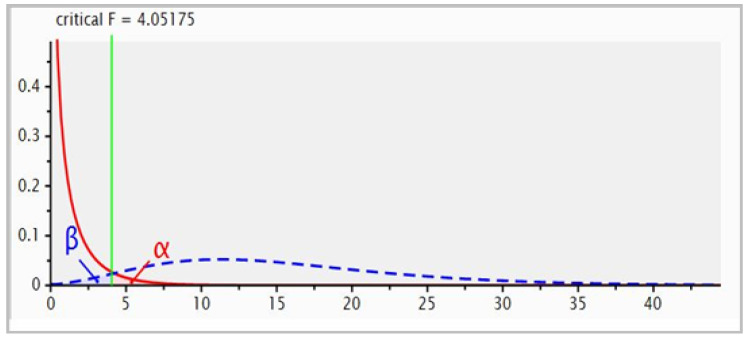
G-Power analysis for statistical power, critical F-value and sample size.

Four hundred 15–18-month-old weaned tropical composite steers of crossbred *Bos indicus* and *Bos taurus* genotypes, weighing 320 ± 21 kg as the initial average liveweight, were utilised in this set-stocked 147-day grazing trial. Prior to the experiment, the steers were grazing on buffel grass-dominated pastures. Experimental steers were randomly assigned to either of the two pastures, buffel grass only (*n* = 200) or mixed buffel grass-*Desmanthus* pastures (*n* = 200) at 2.87 and 2.60 ha/steer stocking rate, respectively, based on the farm manager’s long knowledge of the paddocks’ carrying capacity and remained constant throughout the trial period. Steers were not supplemented throughout backgrounding and were weighed on days 0, 49, 79 and 147 after the onset of grazing. Steers were brought from the paddocks at 09:00 h, left in the holding yards for one hour and weighed between 10:00 h and 14:00 h. Unfasted weights were recorded automatically (Gallagher 65 Scanlon Drive, Epping, Victoria 3076, Australia) and the average daily weight gain (ADG) was calculated by regression using the four weigh points. An *a priori* power analysis using G-Power was conducted to determine the appropriate sample size (Figure 1). A total sample size of 50 steers was required to achieve statistical power of 80% with a critical F-value of 4.0 for a large effect size and a significance level of 0.05. Therefore, twenty-five steers per paddock were randomly selected on day 0 for body condition (BCS) scoring using a five-point scoring system (1–5) [30] and faecal samples taken in parallel with the weighing session. Blood samples were collected from these same 50 steers during days 0 and 147 weighing sessions.

### 2.3. Pasture Sampling and Analysis

The Botanal technique [47] was used, pre- and post-grazing, to estimate pasture yield, botanical composition, ground cover and woody cover [47,48]. Since no substantial pasture growth was expected due to limiting moisture levels throughout the grazing period, grazing utilization was estimated as a percentage of the grazed stock as described by Stoddart [49]. Estimates were made in 0.50 × 0.50 m quadrats assigned on a 100 m × 100 m grid pattern on predetermined GPS points to ensure uniform sampling across the paddocks. The number of quadrats per paddock varied, with paddock size resulting to 595 and 507 quadrats for the buffel-grass-only and mixed buffel grass-*Desmanthus* paddocks, respectively. Representative pasture samples were collected from both paddocks, four times over the course of the experiment; at the beginning, end and twice during grazing. Buffel grass and *Desmanthus* were analysed as they were the dominant pastures, while *Acacia harpophylla* (brigalow) was the dominant woody cover, and steers were observed to browse on its leaves. Although they are palatable, *S. kali*, *U. mosambiencensis* and *Portulaca* spp. were not analysed because their contribution was minimal, below 5% of the pasture botanical composition. Buffel grass and *Desmanthus* samples were harvested by cutting at 5 cm above the ground while brigalow samples consisted of leaves and soft branches approximately 10 cm long. Pasture samples were transported in cooler boxes and stored at −20 °C until being analysed in the laboratory. The samples were oven dried at 60 °C for 48 h, ground to pass through a 1 mm screen using a Cyclotec mill (Foss Tecator AB, Hoganas, Sweden) and analysed for neutral detergent fibre (NDF), acid detergent fibre (ADF), organic matter (OM), crude protein (CP) and dry matter digestibility (DMD). Total nitrogen (N) was determined by the Dumas combustion method using a Leco CN628 N Analyser (Leco, St. Joseph, MI, USA) [50] and CP calculated using total N × 6.25. NDF (without heat-stable α amylase) and ADF concentrations were determined sequentially using an ANKOM 200/220 Fibre Analyser (ANKOM Technology, Fairport, NY, USA) according to the methods of Van Soest et al. [51] and Goering and Van Soest [52], respectively, and hemicellulose was calculated as the difference between NDF and ADF. OM was determined by ashing the samples according to the methods of Faichney and White [53]. In vitro DMD was determined using a modified pepsin-cellulase technique [54] and metabolisable energy (ME) was calculated as DMD × 0.172–1.707 [55].

### 2.4. Faecal Sampling and Analysis

To determine the nutritive value of the diet selected by the steers during grazing, faecal samples were collected from the rectum of 50 steers (25 from each paddock) and from random dung pats in each paddock close to the watering points on weigh days. Faecal samples were transported in a cooler box and stored at −20 °C awaiting laboratory analysis. The samples were dried and ground as previously described for the pasture samples. Faecal near infrared reflectance spectroscopy (FNIRS) (NIRSystems FOSS 6500) as described by Dixon and Coates [56,57] was used to determine CP, DMD, non-grass pasture proportion in the diet (comprising native and sown legumes, forbs and browse) and faecal N at the CSIRO Floreat laboratory (Floreat, WA, Australia). Spectral analyses, data manipulation and spectra calibrations were carried out using ISI (Infrasoft International) software NIRS 3 (Version 3.10, Port Matilda, PA, USA). The calibration equations used were developed for cattle grazing tropical and subtropical pastures [58,59].

### 2.5. Plasma Metabolites Analysis

To assess the steers’ nutritional and health status, blood samples were collected at the start and end of the grazing period from the sample 50 steers by caudal venipuncture into 10 mL heparin-containing BD Vacutainer tubes. Plasma was isolated using a portable horizontal bench-top centrifuge (StatSpin Express 4, Iris Sample Processing, Westwood, MA, USA) at 4000× *g* for 5 min at room temperature. Plasma samples were transferred into labelled 15 mL aliquot tubes and stored at −20 °C pending laboratory analysis. Plasma non-esterified fatty acids (NEFA), beta-hydroxybutyrate (BHB), total bilirubin, creatinine and glucose were analysed using the colorimetric, 3-hydroxybutyrate dehydrogenase, modified diazo, kinetic modified Jaffe and hexokinase methods of the AU480 chemistry analyser (Beckman Coulter, Inc., Brea, CA, USA), respectively, according to the manufacturer’s procedures.

### 2.6. Statistical Analysis

All data were analysed using SAS software version 9.4 (SAS Institute, Cary, NC, USA). Growth performance and blood metabolites data were analysed using the General Linear Model procedure (PROC GLM) analysis of variance with the animal as the experimental unit. Backgrounding pasture, days since onset of grazing and their interactions were fitted as fixed effects, while liveweight (LW), NEFA, BHB, total bilirubin, creatinine and glucose were the dependent variables. The same model was used for the faecal parameters analysis with backgrounding pasture, month and their interactions fitted as fixed effects and faecal N, diet CP, DMD and diet non-grass as the dependent variables. Backgrounding pasture was the only fixed effect for the ADG analysis. Effects were declared significant at *p* ≤ 0.05. Where significant, differences between means were tested by least significant difference (LSD) comparison test. Simple linear regression using the PROC REG was used to determine the relationship between diet non-grass and CP or CP and DMD.

## 3. Results

### 3.1. Rainfall and Pasture Characteristics

Throughout the pasture establishment and grazing periods, the total annual rainfall was below average (598.2 mm/annum) at 421, 368 and 305 mm for the years 2017, 2018 and 2019, respectively (Table 1). The wet season preceding the grazing period commenced in October 2018 and ended in April 2019. The rest of the year was fairly dry, and the next wet season had not started by the time grazing period ended in December 2019.

Table 2 presents DM yield, ground cover, woody cover and the five most dominant pastures species in the two paddocks. Native legumes and forbs such as *Rhynchosia minima*, *Sida* spp., *Convolvulus* spp., *Cleome viscosa* and *Abutilon andrewsianum* were below 0.2%. Buffel grass utilisation in the buffel grass and *Desmanthus* paddocks was 36.5% and 48.7%, respectively, while *Desmanthus* utilisation was 83.5%. Proximate analysis data of the pastures are presented in Table 3. CP was lowest in buffel grass and highest in *Desmanthus*, while DMD and ME were higher in brigalow compared to buffel grass and *Desmanthus*.

### 3.2. Diet Selected during Grazing

Diet CP and DMD were similar throughout the study for the steers on buffel grass, but varied significantly for the steers on *Desmanthus*, with the lowest values recorded on day 49 (Table 4). Faecal N did not vary with backgrounding pasture but reduced significantly by the end of grazing (*p* = 0.001). There was no effect of pasture on the non-grass diet, but a decrease over time (*p* = 0.001) was observed, with the lowest values recorded on day 147. At the beginning of the study, there was no difference in the quality of diet selected by the two groups. The initial diet similarity is indicated by the similar CP, Faecal N, DMD and diet non-grass on day 0. Overall, DMD was higher for the buffel grass than the *Desmanthus* steers (55.5% and 54.2%, respectively; *p* = 0.001).

**Figure 2:**
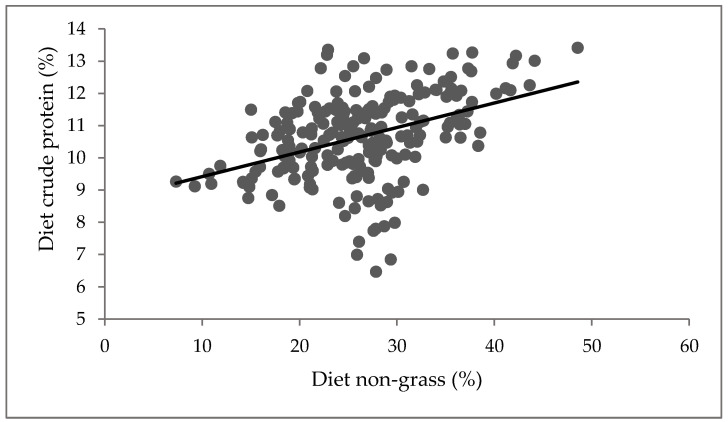
Relationship between diet non-grass and crude protein. Y = 8.66 + 0.076X; where Y = diet crude protein and X = diet non-grass, R^2^ = 0.16, *p* < 0.001.

**Figure 3:**
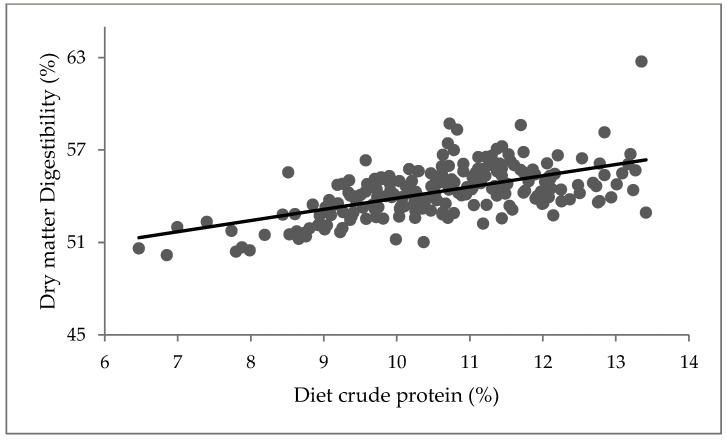
Relationship between diet crude protein and dry matter digestibility Y = 46.60 + 0.728X; where Y = %DMD and X = %CP, R^2^ = 0.34, *p* < 0.001.

A positive relationship was observed between the diet CP and non-grass (Figure 2; *p* < 0.001). CP increased with an increase in diet non-grass component, while DMD increased with an increase in diet CP (Figure 3; *p* < 0.001). However, diet non-grass accounted for only 16% variability in CP, while diet CP accounted for 34% variability in DMD (Figure 3).

### 3.3. Plasma Metabolites

Plasma metabolite data are presented in Table 5. No significant difference in plasma metabolites concentration was observed for steers backgrounded on *Desmanthus*-buffel grass mixed compared to buffel-grass-only pastures, although NEFA tended to be higher for the buffel-grass steers (*p* = 0.058), whereas sampling period had a significant effect on all metabolites except NEFA. Total bilirubin (*p* = 0.041) and glucose (*p* = 0.001) decreased, while BHB (*p* = 0.001) and creatinine (*p* = 0.001) increased for both groups, although the BHB increase in the *Desmanthus* group was not significant. An interaction between period and pasture (*p* = 0.011) was observed for the creatinine with a greater increase observed for the *Desmanthus* than the buffel-grass steers.

### 3.4. Growth Performance

Steer LW, BCS and ADG data are presented in Table 6. Backgrounding pastures did not affect steers’ performance. An increase in LW and BCS was observed throughout the study (*p* < 0.001). Steers’ final LW was 431 and 433 kg, and BCS was 4.1 and 3.9 for the buffel grass and *Desmanthus* paddock steers, respectively.

## 4. Discussion

### 4.1. Pasture Characteristics

The DM yield of the buffel grass pasture in this study (3.4–3.6 ton/ha) was lower than that average reported for the buffel grass pastures in the Brigalow region of Central Queensland (4.5–5.2 ton/ha) [60]. The low yield could be due to the below-average rainfall received during the study period [61]. Although *Desmanthus* contributed a small proportion of initial pasture biomass (11.5%) in the study, pasture DM yield was 443 kg/ha higher in the *Desmanthus* paddock compared to the buffel-grass-only paddock. This finding agrees with other studies that reported an increase in pasture yield when legumes were oversown with grass pastures compared to grass-only pastures in the tropics [62,63]. The presence of 11–33% legumes in temperate pastures was found to increase DM yield, but with a reduced yield benefit as the legume proportion increased to 67% or more [64]. Legumes increase pasture productivity by contributing to increased light capture compared to pure grass stands [65]. Furthermore, nitrogen-fixing legumes promote grass growth by providing nitrogen for the companion grass if moisture is not limiting [66,67].

The CP of *Desmanthus* in this study was lower than that reported for *D. leptophyllus*, *D. virgatus* and *D. bicornutus* grown in a semi-enclosed greenhouse in winter (11.2–18.9%) and spring (13.2–18.2%) seasons [22]. Durmic et al. [68] reported 12.2 to 21% CP in winter and 9.8–19.2% CP in spring. However, one cultivar-*D. virgatus* (Marc) had a CP content of 6.2% in spring. In this study, buffel grass CP was very low (4.4%). The low CP agrees with a review of studies carried out in Central Queensland that reported a decline in buffel grass CP to below 6% in winter [6].

### 4.2. Diet Selected during Grazing

Dietary CP and DMD are the primary limiting factors of growth performance in cattle grazing low-quality pastures in the Australian subtropics during the dry season [69]. Limited CP levels result in below-optimal microbial growth required for structural carbohydrate digestion in the rumen, which in turn depresses feed intake [69,70]. In this study, steers in both paddocks consumed diets with higher CP (8.8–11.6%), DMD (52.1–55.9%) and ME (7.3–7.9 MJ/Kg DM) compared to the CP (4.4–8.5%), DMD (46.9–48.4) and ME (6.8–6.9 MJ/Kg DM) obtained from the pasture proximate analysis. Although, the brigalow DMD and ME were higher at 60.6% and 8.7 MJ/Kg DM, respectively. Ruminants consume diets that differ from the average available biomass in plant species, plant parts and nutrient content [71,72] as a result of foraging behaviour influenced by short-term and long-term decisions, such as which plant to select, how long to search between bites and where to graze [72]. Hence, pasture samples do not adequately represent the diet consumed by grazing animals [73].

It was surprising to observe similar diet non-grass components in the consumed diet of steers in both paddocks. Steers on buffel grass might have consumed non-grass pastures from forbs, native legumes and woody shrubs. Bowen et al. reported 11% C3 forage biomass in cattle grazing C4 perennial-grasses-only pastures and attributed it to naturalised legumes and other dicots present in the pastures [74]. The CP and DMD of selected pastures were lower than those selected by steers grazing the Leucaena-grass pasture (12.4% and 62%, respectively) [57]. However, CP was higher and DMD was similar to that reported for cattle grazing varying perennial grass pastures, forbs and shrubs that consumed a diet with 5.5–8.11% CP and 52.1–55.2% DMD [75]. Although metabolisable protein is a better measure of protein requirement than CP [55], it was not possible to determine the metabolisable protein of the diet selected by steers in this study. Dixon and Coates [76] reported that rumen degradable N is likely to be restrictive only when the DMD: CP ratio exceeds 8 to 10. In the current study, the DMD: CP ratio ranged between 4.8 and 5.9 for both paddocks, indicating that rumen degradable N was not limiting [76].

Non-grass pastures constituted between 17.4–32.4% of the diet consumed. This falls within the range reported for heifers grazing a mixture of Verano and Seca stylos with Sabi grass that selected 15–63% stylo [14]. Among the factors that influence the diet composition of grazing animals are pasture species on offer, availability, palatability and nutritive value of the associated grass [56]. Leucaena in the diet was observed to decline steeply from 87% to 10% with reducing availability during the dry season [57]. In another study, where the entire cattle diet consisted of Mulga (*Acacia aneura*) during the dry season when Mulga was the only available forage, the Mulga proportion reduced to 30% during the wet season when moisture stimulated grass growth [56]. In grass-dominated pastures, cattle consumed 10% non-grass components during the pasture growing season, which increased to over 70% in the dry season [56]. Cattle grazing varying perennial grass pastures, forbs and shrubs consumed 19–49% non-grass components [75]. In the Mitchell-grass-dominated pastures, the non-grass proportion in sheep and cattle diets was high during the wet season and reduced in the dry season. The authors attributed the trend to high palatability of the non-grass pasture species encouraging higher preference when available, but consumption dropped with a decrease in availability during the dry season [56,77]. In grass-dominated pastures consisting of just 2% forbs, cattle consumed up to 15% non-grass during the dry season, indicating high forb selection [56]. Forb and browse are often higher in N and metabolisable energy than grasses, especially when grasses are senesced [78,79]. These studies indicate that cattle can consume large amounts of palatable non-grass pastures when not limited by availability. *Desmanthus* utilisation in the current study was very high (83.5%) suggesting that consumption was limited by availability. Therefore, a higher percentage of *Desmanthus* legume in the pastures may be required for improved non-grass pastures and CP intake to be observed. Thomas [67] suggested that 20–30% DM legume content is required for 10–40% pasture utilisation, and 35–45% DM legume at higher pasture utilisation levels of 50–70% for a productive and sustainable pasture.

### 4.3. Plasma Metabolites

More accurate assessment of nutritional and health status in cattle can be achieved by including plasma metabolites analysis than from BCS and LW alone [30]. The glucose levels were similar to those reported for cattle grazing dormant pastures [80,81] and were within the normal range for beef cattle (2.5 to 5.5 mmol/L) [82,83]. The lack of difference in glucose concurred with results for cattle fed low-quality Sudan grass (*Sorghum* sp.) hay (CP 3.9%) supplemented with soybean alone or with pelleted Silver-grass (*Miscanthus* sp.) to achieve 9.6% CP levels [84]. The decline in glucose from the start to the end of the grazing period is in agreement with results reported in other studies. For instance, rangeland-grazing beef cattle blood glucose decreased from summer, fall, winter to spring [36]. Similarly, a decline in blood glucose was reported for temperate-breed steers during the ‘store’ period [85]. The glucose decline over time can be explained by a decline in feed intake resulting from declining pasture availability [30,36].

Backgrounding pastures did not influence plasma NEFA concentration, indicating that steers were not mobilising body energy reserves in the current study [36,37]. NEFA levels are reported to increase with maturity of forage, which could indicate a negative energy balance [36,37]. In this study, grazing started when the pastures had senesced; hence, no difference in maturity over time was taking place. The increase in BHB levels over time for the buffel-grass steers was unexpected since there was no difference in the NEFA levels. However, this increase was marginal and the plasma BHB level was below a 1.2 mmol/L concentration reported as the threshold to indicate hyperketonemia in cows [86].

Creatinine is produced mainly in the skeletal muscles by the degradation of creatine and creatine phosphate to produce energy [87] and it is commonly associated with renal disorders [88]. Reduced creatinine levels are also indicative of prolonged tissue protein catabolism [30]. In this study, all the steers had creatinine levels within the normal range reported for cows (88.4–177 µmol/L) [87] and bulls (98.7 ± 14.7 µmol/L) [89]. Creatinine levels increased with time for both groups indicating that no catabolism was taking place but rather an increase in muscle mass [30,90].

Bilirubin levels were similar to the normal range reported for extensive range beef cattle [88,89] and the Angoni cattle on grass pastures (2.7 ± 1.4 µmol/L), although the quality of the pasture was not described [89]. Issi et al. [91] reported elevated total bilirubin levels in dairy cows diagnosed with subclinical and clinical ketosis. The authors associated the bilirubin increase with the existence of a functional disorder or liver damage. The similar levels of total bilirubin in the current study may indicate that the caloric intake of steers on both pastures was comparable. It is pertinent to state that going by the plasma metabolite profiles, all the steers in this study were healthy; indicating that, with or without *Desmanthus* inclusion in the diet of grazing steers, animal health status was not compromised.

### 4.4. Growth Performance

The animal growth response to grass pastures oversown with legumes depends on legume yield and quality [46]. Contrary to other studies that reported an increase in LW gain in cattle [26,43], sheep [28,92] and goats [93] supplemented with *Desmanthus* compared to their counterparts fed grass only diets, no difference was observed in this study. This could be due to the lack of increase in diet CP intake in the *Desmanthus* paddock compared to the buffel grass paddock due to low *Desmanthus* levels. An increase in weight gain for cattle supplemented with other tropical legumes has been reported [74,94,95,96]. Zebu steers grazing low-quality standing hay supplemented with 0.8 kg DM Leucaena leaf meal improved daily weight gain from −0.3 to 0.26 kg [97]. Miranda et al. [98] reported a 0.7 kg higher daily weight gain for cattle supplemented with *Stylosanthes guianensis* compared to cattle fed rice straw and *Brachiaria* spp. grass only. Similar to our study, Suybeng et al. [23] reported no difference in LW gain between steers fed Rhodes grass only or supplemented with different levels of *Desmanthus*. The authors attributed the results to low diet ME (6.1–8.2 MJ/Kg DM) and feed intake (1.2–1.6% per Kg LW) that resulted in low daily ME intake (22–39 MJ/Kg DM). In the current study, the selected diet contained at least 7.3 MJ/Kg DM ME, but feed intake could not be determined. Steers in both paddocks had similar weight gain and BCS, and no weight loss was recorded. The finding concurs with a review of eleven studies by Bowman et al. [99] who reported that a pasture diet with CP above 5.6% results in weight gain. Detmann et al. [100] estimated that 10.8 g/kg CP is required to achieve the apparent equilibrium point where the N efficiency of utilisation is nil. A 5.6% CP level was achieved in both paddocks throughout the study while 10.8 g/kg CP failed to be achieved only on days 49 and 147 in the *Desmanthus* paddock. This may indicate that dietary CP in this study was sufficient for rumen microbial growth [9,69,101]. Regardless of the lower dietary CP on days 49 and 147 for the *Desmanthus* steers, no effect on LW was observed. This could be due to the CP and DMD: CP ratio that persisted above 5.6 and 8, respectively [76,99], maintaining sufficient rumen function. Supplementing steers with 15%, 22% and 31% *Desmanthus* was observed to improve rumen function as indicated by the increased total volatile fatty acids concentration in the rumen [23]. Therefore, more studies are required to understand the effect of *Desmanthus* on rumen function.

The ADG of steers grazing buffel-grass-only pastures (0.74 kg/day) was within the 0.2 to >1.0 kg range reported for buffel grass pastures in the Brigalow region of Queensland [6]. However, this is higher than the 0.11 and 0.44 kg/day reported for buffel grass only and buffel grass–*Desmanthus* pastures, respectively, in a similar environment [26], and −0.25 to 0.17 kg/day reported for steers grazing buffel-grass-dominated pastures in the monsoonal climate region of Northern Territory during the dry season [102]. The variance in ADG could be due to differences in stocking rate resulting in varying pasture availability. The stocking rate was 0.55–1.92 ha/steer compared to 2.57 and 3.02 ha/steer in the present study. Individual animal weight gain declines with an increase in stocking rate when not accompanied by increase in pasture biomass due to competition for forage [4].

The main drivers of profitability in grazing systems are annual liveweight gain and the stocking rate [6]. Although the final liveweight for both groups was similar in the current study, the buffel grass–*Desmanthus* mixed paddock had a higher stocking rate compared to the buffel-grass-only paddock by 9.5%. Increasing the stocking rate increases the annual LW per ha [103], promoting profitability [6]. In our study, liveweight gain per hectare was calculated to be 37.8 and 42.4 kg/ha for the buffel and *Desmanthus* pastures, respectively. A strong correlation between the cattle stocking rate and the daily live weight gain (R^2^ ≤ 0.93) was reported for beef cattle grazing grass-dominated pastures with 5–8.1% CP [104]. The authors associated the decline in LW as the stocking rate increased with reduced pasture availability.

Legumes offer the greatest weight gain advantage during the late wet and the dry seasons [14]. This study took place during the dry season only; hence, the response of the steers to *Desmanthus* pastures during the wet and transition seasons was not examined. Cattle grazing buffel grass and *Centrosema brasilianum* (Centro) were observed to select more Centro during the wet to dry transition season than during the dry season at 22.1–40% and 19.7–20.9%, respectively [103]. A similar trend was reported for *Chamaecrista rotundifolia* [105]. However, low nutritive value and palatability of pasture in the seasonally dry subtropics of northern Australia are endemic in the dry season [70,106], hence more controlled pen studies are required to determine the effect of varying levels of *Desmanthus* on the rumen fermentation and growth performance of grazing cattle during the dry season. In addition, previous grazing nutrition is reported to influence the growth performance of cattle during the feedlot finishing phase and carcass traits [107]. Further studies are required to determine the feedlot growth performance and carcass quality of *Desmanthus* backgrounded beef cattle.

## 5. Conclusions

This study evaluated the possibility of using *Desmanthus* legume oversown in Buffel grass pastures to improve growth performance and plasma metabolites profile during the nutrient-limiting dry season in Northern Australia. The results showed no significant effect of *Desmanthus* at low inclusion levels in backgrounding pastures on LW, weight gain and plasma metabolites. Therefore, the hypothesis that backgrounding steers on Buffel grass-Desmanthus mixed pastures would elicit significant changes in plasma glucose, bilirubin, creatinine, non-esterified fatty acids and β-hydroxybutyrate, resulting in higher liveweight gains than in steers on buffel grass only pastures was rejected. Though the lack of difference may be due to the high performance of the buffel grass pastures atypical for the dry season in this region, the main drivers of profitability in grazing systems are annual liveweight gain and stocking rate. The similar weight gain at higher stocking rate indicate that *Desmanthus* may have the potential to improve profitability in the extensive grazing systems of northern Australia and other similar environments by improving pasture carrying capacity. Further research is required to investigate the effect of feedlotting and on-station pen feeding trial with the *Desmanthus* legume to better understand its effect on growth, plasma metabolites, rumen volatile fatty acids, carcass characteristics and meat quality parameters of intramuscular fat content, fat melting point and muscle fatty acid composition in beef cattle. In addition, studies are required to evaluate the growth performance and plasma metabolites of cattle backgrounded on grass pastures oversown with higher levels of *Desmanthus*.

## Figures and Tables

**Table 1:** Monthly and total annual rainfall (mm) for the years 2017, 2018 and 2019.

Year	Jan	Feb	Mar	Apr	May	Jun	Jul	Aug	Sep	Oct	Nov	Dec	Annual Total
2017	68	39	105	6	8	0	22	0	0	107	42	24	421
2018	19	118	36	6	0	24	4	10	4	68	41	38	368
2019	0	1	111	82	3	5	24	15	0	40	20	5	306

**Table 2:** Pasture characteristics of the buffel grass and *Desmanthus* paddocks prior to commencing and at the end of the grazing period. Data presented in percentages unless otherwise stated.

Variable	Buffel Grass Paddock		*Desmanthus* Paddock	
	Pre-Grazing	End of Grazing	Pre-Grazing	End of Grazing
Ground cover	63.7	38.0	68.7	29.7
Woody Cover	0.7	0.7	0.5	0.7
Dry matter yield (kg/ha)				
Total yield	4066	1854	4509	1425
*Cenchrus ciliaris*	3532	1700	3372	1260
*Desmanthus* spp.			502	88.6
*Salsola kali*	8.0	4.2	131	3.9
*Urochloa mosambicensis*	158	57.2	112	28.1
*Portulaca* spp.	18.0	5.5	80.0	2.1
Botanical composition				
*Cenchrus ciliaris*	90.1	91.7	77.2	88.4
*Desmanthus* spp.			11.5	6.2
*Salsola kali*	0.2	0.3	3.0	0.3
*Urochloa mosambicensis*	4.0	3.1	2.6	2.0
*Portulaca* spp.	0.5	0.3	1.8	0.2

**Table 3:** Mean chemical composition and dry matter digestibility (±standard deviation) of buffel grass, *Desmanthus* and brigalow leaves during the backgrounding period. Data are in %DM unless otherwise stated.

Variable	Buffel Grass	*Desmanthus*	Brigalow
DM (%)	84.9 ± 3.1	68.3 ± 3.4	64.6 ± 1.6
Neutral detergent fibre	73.9 ± 1.0	62.8 ± 2.0	38.7 ± 0.5
Acid detergent fibre	43.4 ± 1.1	40.9 ± 1.6	25.5 ± 1.5
Dry matter digestibility	46.9 ± 1.1	48.4 ± 1.2	60.6 ± 1.1
Organic matter	93.1 ± 0.3	94.6 ± 0.5	91.8 ± 0.3
Ash	7.2 ± 0.2	5.4 ± 0.5	8.2 ± 0.3
Hemicellulose	30.5 ± 1.3	21.9 ± 2.0	13.2 ± 2.0
Crude Protein	4.4 ± 0.9	8.5 ± 1.4	7.5 ± 0.3
Metabolizable energy (Mj·kg^−1^ DM) ^1^	6.9 ± 0.1	6.8 ± 0.128	8.7 ± 0.2

DM = dry matter; ^1^ Estimated from in vitro DMD as DM digestibility × 0.172 − 1.707 [55]; MJ = megajoules.

**Table 4:** Effect of pasture backgrounding on dietary CP, DMD, diet non-grass and faecal N as estimated from faecal near infrared reflectance spectroscopy.

Variable	Paddock	Days Since the Onset of Grazing				SEM	*p*-Value		
		0	49	79	147		P	D	P*D
Diet CP (%)	Buffel grass	11.26 ^ab^	11.70 ^a^	10.99 ^ab^	10.92 ^ab^	0.995	0.001	0.001	0.001
	*Desmanthus*	11.28 ^ab^	8.78 ^d^	10.61 ^bc^	9.94 ^c^				
Faecal N (%)	Buffel grass	1.75 ^a^	1.75 ^a^	1.73 ^a^	1.56 ^b^	0.150	0.68	0.001	0.14
	*Desmanthus*	1.74 ^a^	1.73 ^a^	1.79 ^a^	1.49 ^b^				
DMD (%)	Buffel grass	53.94 ^c^	55.11 ^ab^	55.07 ^ab^	55.92 ^a^	1.24	0.001	0.001	0.001
	*Desmanthus*	54.16 ^bc^	52.09 ^d^	54.72 ^bc^	53.83 ^c^				
ME (MJ/Kg DM) ^1^	Buffel grass	7.57 ^c^	7.77 ^ab^	7.76 ^ab^	7.91 ^a^	0.0381	0.001	0.001	0.001
	*Desmanthus*	7.61 ^bc^	7.25 ^d^	7.71 ^bc^	7.55 ^bc^				
DNG (%)	Buffel grass	32.40 ^a^	28.09 ^bc^	26.27 ^c^	19.94 ^d^	4.91	0.65	0.001	0.089
	*Desmanthus*	31.87 ^ab^	27.72 ^c^	28.52 ^abc^	17.37 ^d^				

^abcd^ Means followed by different letters in the same row are significantly different between pastures and days at the *p* < 0.05. ^1^ Estimated from in vitro DMD as DM digestibility × 0.172 − 1.707 [55]; MJ = megajoules; SEM = standard error of the mean; P = paddock; D = sampling day; P*D = paddock and days interaction; CP = crude protein, N = nitrogen; DMD = dry matter digestibility; DNG = diet non-grass.

**Table 5:** Effect of pasture backgrounding on plasma metabolites (LS means).

Metabolite	Pasture	Sampling Period		SEM	*p*-Value		
		Day 0	Day 147		P	D	P*D
Total Bilirubin (µmol/L)	Buffel grass	2.93	2.29	1.21	0.41	0.041	0.094
	*Desmanthus*	2.81	2.30				
BHB (mmol/L)	Buffel grass	0.22	0.28	0.0603	0.35	0.001	0.68
	*Desmanthus*	0.21	0.25				
Creatinine (µmol/L)	Buffel grass	94.48	109.15	16.27	0.12	0.001	0.011
	*Desmanthus*	89.25	122.70				
NEFA (mmol/L)	Buffel grass	0.45	0.36	0.177	0.058	0.21	0.31
	*Desmanthus*	0.36	0.33				
Glucose (mmol/L)	Buffel grass	5.9	4.8	1.00	0.46	0.001	0.40
	*Desmanthus*	5.7	4.8				

BHB = β-hydroxybutyrate; NEFA = non-esterified fatty acids; SEM = standard error of the mean; P = pasture; D = sampling day; P*D = pasture and sampling day interaction.

**Table 6:** LW, ADG and BCS of steers backgrounded on buffel grass alone or with *Desmanthus*.

Variable	Pasture	Days Since the Onset of Grazing				SEM	*p*-Value		
		0	49	79	147		P	Days	P*D
LW (kg)	Buffel grass	319 ^d^	372 ^c^	392 ^b^	431 ^a^	18.9	0.14	0.001	0.21
	*Desmanthus*	322 ^d^	369 ^c^	396 ^b^	433 ^a^				
BCS	Buffel grass	3.46 ^c^	3.60 ^bc^	3.58 ^bc^	4.10 ^a^	0.38	0.51	0.001	0.36
	*Desmanthus*	3.46 ^c^	3.65 ^bc^	3.59 ^bc^	3.90 ^ab^				
Overall ADG (kg/day)	Buffel grass	0.74				0.13	0.78		
	*Desmanthus*	0.75							

^abcd^ Means followed by different letters in the same row are significantly different between pastures and days at the *p* < 0.05. LW = liveweight; ADG = average daily gain; BCS = body condition score; SEM = standard error of the mean; P = pasture; P*D = pasture and days interaction.

## Data Availability

Data available on request.
